# Preliminary Validation of Two Brief Screening Measures for Eating Disorders in Adults with Chronic Pain

**DOI:** 10.1007/s10880-025-10111-2

**Published:** 2025-12-28

**Authors:** Lindsay G. Flegge, Michelle L. Miller, Amy E. Williams, Brianna L. Jehl, Michael A. Bushey

**Affiliations:** 1 Department of Psychiatry, Indiana University School of Medicine, Indianapolis, United States; 2 Indiana University Health, Indianapolis, United States

**Keywords:** Chronic pain, Disordered eating, Screening tools, EAT-8, EDE-Q8

## Abstract

Chronic pain and eating problems frequently co-occur, with disordered eating behaviors such as emotional eating, food restriction, and appetite fluctuation contributing to adverse outcomes. However, there are no validated screening tools specifically designed for assessing eating problems in chronic pain populations. This study aimed to validate two brief eating disorder measures, the Eating Attitudes Test-8 (EAT-8) and Eating Disorder Examination-Questionnaire-8 (EDE-Q8), for English-speaking adults presenting for chronic pain treatment. Participants included 173 adults seeking treatment at a pain evaluation service. Participants completed the EAT-8 and EDE-Q8 alongside self-report clinical measures. Demographic and clinical data were extracted from medical records. Statistical analyses assessed internal reliability, construct validity, and differences in eating disorder measures by BMI and eating disorder history. Both the EAT-8 and EDE-Q8 demonstrated acceptable internal reliability (McDonald’s omega ≥ 0.7) and strong construct validity, with significant correlation between the two measures (*r* = 0.77). Scores varied significantly by BMI and eating disorder history, with higher scores observed among participants with obese BMI or a history of eating disorders. However, less than 20% of participants felt the measures fully captured their experiences, suggesting potential gaps in assessing eating problems specific to chronic pain. The EAT-8 and EDE-Q8 are valid and reliable tools for identifying disordered eating behaviors in chronic pain patients. However, these measures may not capture the full spectrum of eating problems unique to this population. Future research should refine screening tools to better assess eating behaviors as they relate to chronic pain.

**Supplementary Information:**

The online version contains supplementary material available at 10.1007/s10880-025-10111-2.

## Introduction

The United States is experiencing a crisis of high impact chronic pain and eating problems. Chronic pain impacts multiple domains of functioning, including physical health, emotional health, sleep, school and work attendance/performance, engagement in social and recreational activities, family cohesion, eating behaviors, and nutrition.(Relieving Pain in America: A Blueprint for Transforming Prevention, Care & Education &and Research. PubMed.doi:^10^., 17226/13172 xxxx; Sim et al., 2021) Chronic pain is also associated with anhedonia and decreased motivation that can precede disruptive eating behaviors and obesity in chronic pain patients.(Lin et al., 2022) A recent study demonstrated 70% of adult patients presenting for treatment of chronic pain reported at least one symptom associated with eating problems, including symptoms that go beyond a formal eating disorder diagnosis such as fluctuating appetite, emotional and stress eating, and eating problems related to medications (Flegge et al., xxxx).

Reported eating problems may not always meet criteria for an eating disorder diagnosis, such as anorexia nervosa, bulimia nervosa, or binge eating disorder,(Lego et al., 2024) however, chronic pain and eating disorders frequently co-occur, share several demographic, temperamental, and biopsychosocial risk factors. Additionally, it has been theorized that eating disorders and chronic pain may share a pathogenesis mechanism of central sensitization (Sim et al., 2021). Central sensitization is a known mechanism contributing to chronic pain, wherein the central nervous system is in a state of increased reactivity leading to pain facilitation and increased risk for chronic pain (Woolf, 2011) Sim et al. (2021) (Sim et al., 2021) hypothesized that central sensitization plays a role in the development of eating disorders as evidenced by frequent somatic complaints, nausea, and abdominal pain with eating among patients with eating disorders. Supporting the potential role of central sensitization in eating disorders, Zucker et al. (2013) (Zucker et al., 2013) found that body image disturbance in patients with anorexia was associated with reported sensory sensitivity and avoidance of sensory experiences. Khalsa et al. (2015) (Khalsa et al., 2015) found that patients with anorexia had increased interoceptive awareness compared to health controls. Although research is needed to confirm the role of central sensitization in eating disorder pathogenesis, the hypothesis highlights the importance of further research evaluating the comorbidities between pain and eating symptoms.

Early detection of eating problems in the chronic pain population is essential for early intervention. Prior research illustrates a pattern of frequent comorbid chronic pain and eating problems, such as pain impacting hunger, nausea, or food preferences (Meleger et al., 2014; O’Loughlin & Newton-John, 2019). Research indicates that it is common for chronic pain patients to avoid eating due to increased pain after eating (Sim et al., 2021). Additionally chronic pain patients are commonly advised to start specific, often restrictive, diets to help manage their pain, which can lead to hypervigilance about their eating behaviors (Sim et al., 2021). However, research involving eating problems among adults presenting for chronic pain treatment is very limited and has focused on gastrointestinal pain disorders (Satherley et al., 2015) or obesity (Narouze & Souzdalnitski, 2015; O’Loughlin & Newton-John, 2019). Chronic pain can often mask or overlap with symptoms of eating problems, making it harder to identify these conditions without reliable screening tools. To date, no specific screening measures exist for assessing eating problems within the context of chronic pain, and it is not known whether existing eating disorder screeners are sufficient for assessment of the wide range of eating problems associated with chronic pain, including eating problems that are specifically related to having chronic pain, eating problems that may be related to chronic pain, or eating problems that co-exist with chronic pain. Validated brief screening measures are essential to inform research and clinical practice decisions. To remedy this, we sought to validate two brief eating disorder measures among patients presenting for treatment of chronic pain.

There is a gap in the literature in validating brief, psychometrically sound screeners for patients with chronic pain. The Eating Attitudes Test (EAT) and the Eating Disorder Examination-Questionnaire (EDE-Q) are both well-established self-report questionnaires for comprehensive assessment of eating disorder psychopathology (Cooper & Fairburn, 1987; Garner et al., 1982) However, they consist of many items (22 and 40, respectively) which makes them ill-suited for patients presenting to a clinical setting for pain management treatment where assessment burden is already a concern. Development of the EAT-8 and EDE-Q8 was a welcome addition to the assessment landscape with initial psychometric results demonstrating excellent item characteristics, very good reliability, and very good model fit for the factorial structure (*N* = 2527: (Richter et al., 2016) *N* = 2508; (Kliem et al., 2016)) Furthermore, there was strong correlation between the EDE-Q8 and the Eating Attitudes Test (EAT). Multiple replication studies also exist which investigate the psychometric properties of these brief versions and have consistently demonstrated that the EAT-8 and EDE-Q8 can be used as standalone screening instruments to aid in reducing administrative and participant burden (Machado et al., 2020a) However, the German version of the EAT-8 and EDE-Q8 was used for initial validation with an age range of 14–92 and the measure was presented to a sample of the population of Germany without a comorbid chronic condition in German, suggesting unknown applicability to English-speaking patients presenting with chronic pain. Although these two measures are correlated, they do have some differences in content assessed. For example the EAT-8, but not the EDE-Q8, has a question asking about fear of being overweight. It is unknown whether one of these measures would be more relevant to a population with chronic pain.

Additional psychometric studies are needed before making either the EDE-Q8 or EAT-8 part of routine research or clinical screening for adult patients with chronic pain to ensure they accurately capture eating problems specific to these patients. The aim of the present study is to validate English versions of the EDE-Q8 and EAT-8 for adult patients presenting for chronic pain treatment. Validating these measures ensures clinicians can promptly identify at-risk individuals, tailor interventions, and address the complex interaction between chronic pain and eating disorder symptoms, hence improving patient outcomes. Furthermore, properly validated tools will facilitate better integration of psychological and physical health for patients, likely making interventions more effective.

## Methods

### Participants

Approval for this study was granted by [Anonymized] Institutional Review Board (IRB). Participants in this study included 173 adults who presented for treatment at Pain Navigation Service (PNS). PNS provides multidisciplinary evaluations and referrals for patients with complex chronic pain conditions at an academic medical setting in the Midwest. Referral sources for these services were diverse, including providers from primary care, physical medicine and rehabilitation, neurology, and sports medicine. New patients received a flyer with study information and link to informed consent. Additionally, previous patients who had consented to be contacted for future research studies were emailed the research prompt with study information.

### Procedure

As part of routine clinical care, patients complete electronic questionnaires assessing pain, functioning, and emotional distress at the time of initial evaluation. For this research study, patients who were 1) 18 and older and 2) spoke English received an electronic prompt on the tablet at the end of these clinical questionnaires asking if they would like to complete an additional two questionnaires for a research study. Consenting participants were asked to complete the EDE-Q8 and EAT-8 along with follow-up questions asking current height, weight, history of eating disorder diagnoses, a Likert scale response question, and a free-response question asking if participants thought the EDE-Q8 and EAT-8 captured their experience with chronic pain and eating. The free-response question specifically asked participants to provide feedback on the EDE-Q8 and EAT-8, so there is minimal concern that participants were providing feedback on the whole battery. Previous patients who had consented to be contacted for future research studies were emailed the same electronic prompt, along with routine clinical questionnaires. Study data were collected and managed using REDCap electronic data capture tools hosted at Indiana University. REDCap (Research Electronic Data Capture) is a secure, web-based software platform designed to support data capture for research studies (Harris et al., 2009, 2019).

## Measures

All measures below were self-report. Demographics (age, sex, gender, and race/ethnicity) and non-eating disorder measures were extracted from the electronic medical record. Only measures completed within 2 weeks of completing the eating disorder measures were included for analysis.

### Eating Behavior Specific Measures

The Eating Disorder Examination-Questionnaire 8 (EDE-Q8) (Kliem et al., 2016) is a shortened version (8-item) of the widely used Eating Disorder Examination Questionnaire (EDE-Q). The EDE-Q8 was used to assess the core symptoms of eating disorders, including preoccupation with weight, shape concerns, and eating behavior*.* Participants are asked about behaviors they engaged in over the past 14 days (e.g., *Have you been trying to cut down on food to control your weight or shape?”)* with a 7-point Likert scale used to rate how common the behavior was (0 = *No days*, 6 = *Every day*). Participants were also asked to use a 7-point scale (*0* = *Not at all to 6* = *Markedly*) to describe their feelings around their weight and body (e.g., “*How unhappy have you felt about your weight?”*). Higher scores indicate more eating pathology with a clinical cutoff of 3.88 (sensitivity = 0.58, specificity = 0.90) (Machado et al., 2020b). The EDE-Q8 maintains the psychometric integrity of the original EDE-Q while offering a more efficient and accessible format for clinical and research settings. In our sample, internal consistency was excellent (McDonald’s omega (ω) = 0.91).

The Eating Attitudes Test-8 (EAT-8) (Richter et al., 2016) is a condensed version (8-item) of the original Eating Attitudes Test (EAT-26) used to quickly assess eating disorder symptoms, including restrictive dieting, preoccupation with food, and body image disturbances. A dichotomized response format is used (0 = *I disagree somewhat* or 1 = *I agree somewhat*) for a score that can range 0 to 8. Scores are classified as low risk vs high risk; a more conservative score of ≥ 3 (sensitivity 67.6 (63.3–71.7); 88.0 (85.7–90.1)) for females and ≥ 2 for males (sensitivity 64.4 (58.6–69.9); 86.2 (83.8–88.4)) classifies the participant as being at high risk of having/developing clinical eating disorders. The EAT-8 is a brief and effective tool for initial screenings in both clinical and non-clinical populations that allows for efficient identification of at-risk individuals to inform further evaluation and intervention treatment. Sample items assess behaviors (e.g., *I eat diet food)* and cognitions (e.g., *I am preoccupied with a desire to be thinner).* Reliability, convergent validity, and score norms by age group have been demonstrated in a German sample (Richter et al., 2016). Internal consistency was acceptable in our sample (ω = 0.76).

After completing these measures, participants were asked, “How well do you think those questions captured your experience with chronic pain and eating?” on a 5-point Likert scale (Not at all—To a Great Extent) and then asked a follow-up open-ended question, “What else do you think is important for us to understand about chronic pain and eating?”.

### Pain Measures

The PEG (Pain, Enjoyment, and General Activity) is a brief, three-item outcome measure that evaluates pain severity, interference with enjoyment of life, and interference with general activity (Krebs et al., 2009). Each item is scored 0–10 and the three items are averaged with larger scores reflecting a higher pain burden. Internal consistency was good in our sample (ω = 0.87).

The Pain Catastrophizing Scale (PCS) is a 13-item measure that evaluates both emotional and cognitive aspects of pain via ratings of catastrophic thoughts and feelings about pain such as magnification, rumination, and helplessness (Sullivan et al., 1995). Items are scored from 0 to 4, with total scores ranging from 0 to 52. Higher scores are indicative of increased pain catastrophizing. Internal consistency was excellent in our sample (ω = 0.95).

### Psychopathology & Functioning Measures

The Patient Health Questionnaire-9 (PHQ-9) is a nine question measure that utilizes the Diagnostic and Statistical Manual of Mental Disorder symptomatology to measure depression severity (DSM-5) (Kroenke et al., 2001) Each item is scored 0–3, with total scores ranging 0–27. High scores are indicative of an increase in symptoms of depression. The PHQ-2 utilizes the first 2 questions of the PHQ-9 and branched logic, such that patients who score ≥ 2 on the PHQ-2 are directed to answer the additional PHQ-9 questions for clarity of depressive symptoms. Internal consistency was acceptable in our sample (ω = 0.75).

The Generalized Anxiety Disorder 7 (GAD-7) is a seven question measure used to assess the severity of generalized anxiety symptoms experienced over the past two weeks (Spitzer et al., 2006). Items are scored from 0 to 3, with total scores ranging from 0 to 21. Higher scores are indicative of greater anxiety symptoms. The GAD-2 utilizes the first 2 questions of the GAD-7 and branched logic, such that patients who ≥ 2 on the GAD-2 are directed to answer the additional GAD-7 questions for clarity of anxiety symptoms. Internal consistency was good in our sample (ω = 0.89).

The Insomnia Severity Index (ISI) is a seven-item measure of insomnia symptoms (Insomnia Severity Index, 2025). Items are scored from 0 to 4, with total scores ranging from 0 to 28. Higher scores are indicative of more severe symptoms of insomnia. Internal consistency was good in our sample (ω = 0.89).

The Patient-Reported Outcomes Measurement Information System (PROMIS) Physical Functioning scale 12a (PROMIS-PF) is a 12-item measure that assesses an individual’s ability to perform physical activities ranging from basic self-care to more demanding tasks (Cella et al., 2010) It evaluates physical capabilities without focusing on performance in specific contexts or conditions. Raw scores are converted to T-scores utilizing tables available from PROMIS at http://www.healthmeasures.net. Internal consistency was excellent in our sample (ω = 0.90).

The Patient-Reported Outcomes Measurement Information System (PROMIS) Social Roles & Abilities (PROMIS-SF) is an 8-item measure that assesses an individual's perceptions of their abilities and limitations in effectively fulfilling social roles (Cella et al., 2010). Items are scored from 1 to 5, with total scores ranging 8–40. Higher scores are indicative of an increased ability to participate in those roles. Raw scores are converted to T-scores utilizing tables available from PROMIS at http://www.healthmeasures.net. Internal consistency was excellent in our sample (ω = 0.96).

## Statistical Analyses

SPSS version 29 was used for all analyses. The EDE-Q8 scale was not completed by 2 participants. Of scales with data collected, overall missingness of single items was 0.6%. Data from one additional EDE-8 and two PROMIS-PF scales were excluded due to > 75% item missingness. For all other missing items, values were imputed using the overall mean score for that item among the entire sample. Summary statistics calculated and reported for each scale include minimum, maximum, mean, standard deviation (SD), skewness, and kurtosis. For all scales, skewness was between -1 and 1 and distributions were treated as normal. Floor and ceiling effects were calculated by measuring the percentage of responses that were the minimum and maximum possible scale values, respectively. To assess construct validity for eating measures, two separate analyses were conducted. The first divided the sample by eating disorder history based on patient response into “yes”, “no” and “not sure” groups. The second divided patients into BMI categories (“underweight”, “healthy”, “overweight”, and “obese”) based on self-report height and weight. For both analyses, one-way ANOVA was conducted to compare between-group means of eating measures and other available clinical measures completed proximally (< 14 days apart from eating scales): PEG, PCS, PHQ-2/9, GAD-2/7, PROMIS-PF&SF, ISI. The assumption of homogeneneity of variances was tested and confirmed (Levene’s statistic not significant at alpha < .05). Tukey’s Honestly Significant Difference (HSD) test was used to conduct post hoc pairwise comparisons for any statistically significant ANOVA results. For construct validity analyses, Pearson correlations were computed between scales. We expected to find high correlation (> 0.5) between the two eating measures, and low-to-moderate (< 0.5) between these measures and other health measures. Response frequencies were calculated for the question about experience capture, and between-group comparisons made by one-way ANOVA. The study size of 173 participants was chosen as this sample size would provide 80% power at a significance threshold of α < .05 to detect at least moderate effect sizes (η^2^ > 0.06) for the one-way ANOVA analyses of the eating measures. This sample size also reflects a > 20 participant to item ratio for the psychometric analyses of the eating measures. While the number of participants who completed the non-eating measures was lower, we still had > 80% power at a significance threshold of α < .05 to detect large effect sizes (η^2^ > 0.06) for the one-way ANOVA analyses for these measures and a > 5 participant to item ratio for all measures.

## Results

### Sample Description

Participants self-reported date of birth, height, and weight. Additional demographic information was extracted from the medical record. The mean (SD) age of our overall sample (*N* = 173) was 45.8 (14.10) years. There were 73 female participants (76%), 81 participants identified as White (85.3%), 86 identified as straight (sexual orientation; 75.4%), 55 were married (48.7%), 31 employed full-time (27%), and most (*n* = 84; 73.7%) had pain for at least 5 years. Participants who provided height/weight information (*n* = 167) reported a mean BMI of 31.8 (SD = 9.5). Demographic variables were comparable between patients who reported a history of eating disorder vs those who did not (Table SI), or across BMI categories (Table S2).

## Scale Properties

Internal reliability for all scales was acceptable (McDonald’s omega ≥ 0.7; Table 1) while reliability for EDE-8, PCS, PROMIS-PF, and PROMIS-SF were excellent (McDonald’s omega ≥ 0.9). As expected, floor effects were most pronounced for PHQ-2/9 and GAD2/7 due to their branched logic structure. While no scale had ceiling effects above 10%, the EAT-8 had the highest ceiling effect (7.6%), compared to 1.2% for the EDE-8.

**Table 1:** Scale properties

*Measure*	*N*	*McDonald’s*	*Minimum*	*Maximum*	*Floor*	*Ceiling*	*Mean*	*SD*	*Skewness*	*Kurtosis*
EAT-8	172	.76	0	8	7.6%	7.6%	4.10	2.40	− 0.03	− 1.11
EDE-Q8	170	.91	0	48	2.9%	1.2%	24.90	14.30	− 0.27	− 1.25
PEG	81	.87	2	30	0.0%	1.2%	18.00	6.30	− 0.38	− 0.28
PCS	79	.95	1	49	0.0%	0.0%	21.10	12.40	0.53	− 0.75
PROMIS-PF	77	.90	25.1	55.8	0.0%	0.0%	36.50	5.90	0.58	0.91
PROMIS-SF	81	.96	25.9	56.8	7.4%	0.0%	39.10	6.90	0.08	− 0.04
PHQ-2/9	81	.75	0	22	37.0%	0.0%	6.40	7.40	0.67	− 1.05
GAD-2/7	81	.89	0	21	21.0%	1.2%	7.10	6.70	0.56	− 0.98
ISI	81	.89	0	28	1.2%	1.2%	12.60	6.60	0.12	− 0.8

*EAT*-8 eating attitudes test-8, *EDE-Q8* eating disorder examination‐questionnaire-8, *PEG* pain enjoyment general activity scale, *PCS* pain catastrophizing scale, *PROMIS* patient-reported outcome measure information system, *PF* physical function, *SF* social function, *PHQ-2/9* patient health questionnaire depression screener/questionnaire, *GAD*-2/7 generalized anxiety disorder screener/questionnaire, *ISI* insomnia severity index

## Construct Validity

When comparing the sample by self-reported eating disorder (ED) history, mean (SD) scores of both EAT-8 and EDE-Q8 differed by category (Table 2). One-way ANOVA revealed a significant difference in EAT-8 scores across ED history groups (*F*(3, 162) = [6.035], *p* = .003), with post hoc pairwise comparisons (Tukey’s HSD test) indicating significant differences specifically between participants with no reported eating disorder history (M = 3.8; SD = 2.3) and for participants with a reported ED history (*M* = 5.9; SD = 2.2, *p* = .003, 95% C.I. = [− 3.5 to − 0.6]). There was also a significant difference in EDE-Q8 scores across ED groups (F(2,162) = [3.255], *P* = .04), with post hoc Tukey’s HSD Test finding significant differences specifically between participants with no reported ED history (*M* = 23.4; SD = 14.1) and with a reported ED history (*M* = 32.6; SD = 14.1)( *P* = .04, 95% C.I. = [− 18.1 to -0.4]. Other clinical measures were not significantly different between ED history groups. When comparing the sample by BMI category, mean (SD) scores of both EAT-8 and EDE-Q8 differed by category (Table 3). One-way ANOVA revealed a significant difference in EAT-8 scores across BMI categories (F(3,162) = [11.816], *P* < .001). Tukey’s HSD found significant differences between underweight (M = 1.3; SD = 2.4) and overweight (*M* = 3.9; SD = 2.3)(*P* = .040, 95% C.I. = [− 5.1 to − 0.1], between underweight and obese (M = 4.9; SD = 2.2)(*P* = .001, 95% C.I. = [− 5.9 to − 1.1], and between healthy (*M* = 2.7; SD = 2.1) and obese (*P* < .001, 95% C.I. = [− 3.3 to − 1.1]. There was also a significant difference in EDE-Q8 scores across BMI categories (F(3, 162) = [25.517], *P* < .001). Tukey’s HSD found significant differences between underweight (M = 10.0; SD = 14.5) and obese (*M* = 31.7; SD = 10.6)(*P* < .001, 95% C.I. = [− 34.7 to − 8.7]), between healthy (*M* = 13.1; SD = 12.0) and overweight (M = 23.0; SD = 13.7) (*P* = .002, 95% C.I. = [− 16.9 to − 2.9]), between healthy and obese (*P* < .001, 95% C.I. = [24.6 to − 12.7]),), and between overweight and obese (*P* = .001, 95% C.I. = [− 14.8 to − 2.7]). Other clinical measures were not significantly different between BMI groups.

**Table 2:** One-way ANOVA comparing prior eating disorder history

*Measure*	*No ED Hx*		*ED Hx ("yes ")*		*“Not sure”*		*p-value*	*Effect size*
	*N*	*Mean (SD)*	*N*	*Mean (SD)*	*N*	*Mean (SD)*		*(*η^2^)
EAT-8	136	3.8 (2.3)	16	5.9 (2.2)	13	4.5 (2.7)	0.003	0.069
EDE-Q8	136	23.4 (14.1)	16	32.6 (14.1)	13	27 (14.6)	0.040	0.039
Pain (PEG)	68	17.6 (6.5)	6	21.3 (4.5)	5	16.6 (4)	0.340	0.028
Catastrophizing (PCS)	66	20.1 (12.3)	6	27.2 (14.8)	5	26.2 (11.6)	0.270	0.035
Physical Function (PROMIS-PF)	65	36.8 (6.2)	6	34.7 (3.2)	5	34.9 (5.1)	0.580	0.015
Social Function (PROMIS-SF)	68	39.7 (6.9)	6	33.5 (5.3)	5	40 (6)	0.100	0.058
Depression (PHQ-2/9)	68	6.1 (7.3)	6	8.3 (9.1)	5	9.5 (8.9)	0.520	0.017
Anxiety (GAD-2/7)	68	6.6 (6.5)	6	11.8 (6.1)	5	9 (9)	0.150	0.048
Insomnia (ISI)	68	12.6 (6.7)	6	16.3 (6.3)	5	10.6 (4.8)	0.320	0.030

*EAT*-8 eating attitudes test-8, *EDE*-Q8 eating disorder examination‐questionnaire-8, *PEG* pain, enjoyment general activity scale *PCS* pain catastrophizing scale, *PROMIS* patient-reported outcome measure information system, *PF* physical function, *SF* social function, *PHQ*-2/9 patient health questionnaire depression screener/questionnaire, *GAD*-2/7 generalized anxiety disorder screener/questionnaire*, ISI* insomnia severity index

**Table 3:** One-Way ANOVA Comparing Scores for BMI Categories

*Measure*	*Underweight*	*Healthy*	*Overweight*	*Obese*	*p-value*	*Effect size*
	*n* = 6	*n* = 39	*n* = 38	*n* = 83		*(*η^2^)
EAT-8	1.3 (2.4)	2.7 (2.1)	3.9 (2.3)	4.9 (2.2)	< .001	0.180
EDE-Q8	10 (14.5)	13.1 (12)	23 (13.7)	31.7 (10.6)	< .001	0.321
	*n* = 2	*n* = 18	*n* = 16	*n* = 44		
PEG	5.8 (0.2)	6.2 (2.3)	4.8 (2)	6.3 (1.9)	0.070	0.084
PCS	36.5 (13.4)	21.8 (14.3)^a^	21.8 (14.4)^c^	19.6 (10.7)	0.290	0.049
PROMIS-PF	39.7 (5.4)	38.6 (7.4)^b^	37.7 (3.6)^c^	35.1 (5.7)	0.130	0.074
PROMIS-SF	42.6 (2.1)	39.2 (7.7)	40.8 (5.9)	38.5 (6.9)	0.630	0.023
PHQ-2/9	8 (9.9)	7.6 (8.1)	6.8 (7.4)	5.9 (7.3)	0.850	0.010
GAD-2/7	7 (2.8)	7.8 (7.1)	7.5 (7.3)	6.8 (6.6)	0.960	0.004
ISI	14.5 (7.8)	14.7 (7.8)	12.2 (5.5)	12 (6.4)	0.490	0.031

*EAT*-8 eating attitudes test-8, *EDE*-Q8 eating disorder examination‐questionnaire-8, *PEG* pain enjoyment general activity scale, *PCS* pain catastrophizing scale, *PROMIS* patient-reported outcome measure information system, *PF Physical Function*, *SF* social function, *PHQ*-2/9 patient health questionnaire depression screener/questionnaire, *GAD*-2/7 generalized anxiety disorder screener/questionnaire, *ISI* insomnia severity index ^a^*N* = 17, ^b^*N* = 16, ^c^*N* = 43

The mean (SD) lag between completion of the eating scales and clinical scales was 4.7 (4.3) days, with *n* = 14 (17%) completed the same day, and *n* = 75 (93%) completed within 8 days. Pearson correlation results are shown in Table 4. As expected, the two eating disorder scales had a strong correlation (*r* = 0.77), while correlations between eating measures and other clinical measures were lower (*r* = − 0.36 to 0.35). Other scale correlations (e.g., moderate to strong correlations between pain, catastrophizing, depression, and anxiety measures) were as expected.

**Table 4:** Pearson correlations

	EAT-8	EDE-8	PEG	PCS	PR-PF	PR-SF	PHQ2/9	GAD2/7	ISI
EAT-8									
EDE-8	.77								
Pain (PEG)	.19	.35							
Catastrophizing (PCS)	.12	.15	.60						
Physical Function (PROMIS-PF)	− .09	− .22	− .53	− .20					
Social Function (PROMIS-SF)	− .30	− .36	− .57	− .39	.60				
Depression (PHQ-2/9)	.09	.14	.52	.74	− .11	− .29			
Anxiety (GAD-2/7)	.16	.18	.39	.65	− .08	− .26	.71		
Insomnia (ISI)	− .02	.03	.42	.37	− .24	− .33	.49	.57	

*EAT*-8 eating attitudes test-8, *EDE*-Q8 eating disorder examination‐questionnaire-8, *PEG* pain, enjoyment, general activity scale, *PCS* pain catastrophizing scale, *PROMIS* patient-reported outcome measure information system, *PF* physical function, *SF* social function, *PHQ*-2/9 patient health questionnaire depression screener/questionnaire, *GAD*-2/7 generalized anxiety disorder screener/questionnaire, *ISI* insomnia severity index

## Scale Feedback Summary

Of the 165 respondents who answered the question, “How well do you think those questions captured your experience with chronic pain and eating?”, *n* = 23 (21.2%) responded “not at all”, *n* = 41 (24.8%) responded “very little, *n* = 60 (36.4%) responded “somewhat”, *n* = 23 (13.9%) responded “quite a lot”, and *n* = 6 (3.6%) responded “to a great extent”. When comparing mean feedback score (with responses coded 1–5) with one-way ANOVA, responses did not differ significantly by ED history (*p* = .48) or BMI category (*p* = .10).

## Discussion

The present study sought to validate two brief eating disorder screens for use in English-speaking patients seeking treatment for chronic pain. This study demonstrated the construct validity of both the EAT-8 and EDE-Q8 in a moderately sized sample. Participants’ responses on both the EAT-8 and EDE-Q8 differed by eating disorder history as well as BMI. As expected, participants with no eating disorder history had the lowest scores, while the participants with an eating disorder history had the highest scores on both measures. Similarly, scores on both measures were lowest for those with an underweight BMI and highest for those with an obese BMI, similar to previously demonstrated associations between screen-based disordered eating and BMI (Alhaj et al., 2022) Other clinical measures were not significantly different between eating disorder history groups or BMI groups. The two eating disorder scales also had a strong correlation, while correlations between eating measures and other clinical measures were low. Of note, there was good internal reliability for the eating behavior measures. This study suggests that both the EAT-8 and EDE-Q8 are valid measures for English-speaking adult patients presenting for chronic pain treatment.

This study adds to the emerging research that has demonstrated strong associations between disordered eating behaviors and chronic pain, often resulting in adverse outcomes (Khalsa et al., 2015; Meleger et al., 2014; Narouze & Souzdalnitski, 2015; O’Loughlin & Newton-John, 2019; Satherley et al., 2015; Sim et al., 2021; Zucker et al., 2013) For example in a previous study, when examining the association between chronic pain and pain-induced comfort eating or overeating pleasurable foods to cope with pain, results showed that over three-quarters of their sample reported engaging in pain-induced comfort eating (O’Loughlin & Newton-John, 2019) Further, pain-induced comfort eating significantly predicted increased BMI, and BMI in turn significantly predicted greater chronic pain interference. Increasing our arsenal of reliable and valid measures of disordered eating behaviors will allow us to better identify signs of poor coping strategies that are being utilized among patients with chronic pain, allowing an opportunity to intervene and reduce poor outcomes (e.g., more pain interference).

Furthermore, others have proposed that eating disorders may also be associated with a pathogenic mechanism of central sensitization (Sim et al., 2021) Central sensitization is a known mechanism contributing to chronic pain, wherein the central nervous system is in a state of increased reactivity leading to pain facilitation and increased risk for chronic pain (Woolf, 2011) Although we are unaware of research directly assessing central sensitization in eating disorders, this theory warrants additional evaluation given the high comorbidity between chronic pain and eating disorders, and similar risk factors for each. We also sought to understand if these scales were accurately capturing chronic pain patients’ experience with disordered eating behaviors. While about 1 in 6 indicated that the survey accurately captured their experience, a little under half (46%) noted that the survey captured their experience not at all/very little. Importantly, responses didn’t differ by eating disorder history or BMI category. This suggests that there may still be some aspects of disordered eating, especially in the context of chronic pain that the current measures are missing. More research is needed for measures that can identify how disordered eating may look, how disordered eating may be utilized as maladaptive coping strategies, and how these behaviors affects various dimensions of chronic pain (Lamoureux & Pagé, 2024) Future research could benefit by utilizing qualitative methods to more comprehensively understand patients’ experiences that are missing from the field’s current measures.

## Clinical Implications

There is not currently a standard of care for how to address eating problems in the context of chronic pain treatment. Symptoms and behavioral characteristics associated with chronic pain can often mimic those of eating disorders (e.g., emotional eating, food restriction, perfectionism), making it harder to identify these conditions without reliable screening tools. Part of the difficulty associated with screening for eating disorders is that there are not universal recommendations for screening in the adult population (Davidson & Barry, 2022) making it more difficult to change norms around screening for specialty clinics and populations. Further, training regarding eating disorders is often limited or absent in primary care where chronic pain patients may first or more often present, compared to greater training within psychiatric training programs (Mahr et al., 2015). In a study seeking to understand training currently present for medical students, only six percent of the 637 surveyed programs incorporated formal rotations focusing on eating disorders (Mahr et al., 2015). This is problematic because physicians are not being trained in how to identify, evaluate, and treat eating disorders effectively, even in the absence of the increased complexity in the presence of comorbid chronic pain.

## Limitations

The present study had several limitations. First, only self-report measures were utilized rather than a clinical interview. Thus, it is unknown how many chronic pain participants also met criteria for any type of eating disorder. Additionally, the non-eating disorder measures were collected as part of routine clinical care, resulting in both a relatively low number of completed measures and lag time between eating disorder measures and other measure collection. Relatively, the sample size of participants in the eating disorder and underweight groups is small and may weaken the generalizability of our findings. Replication with a larger sample of those with comorbid eating disorders and chronic pain is needed. Additionally, future research should utilize a full clinical interview to determine if clinician administered measures more accurately identify eating disorders in chronic pain populations. Secondly, participants were mostly straight White females, resulting in less diversity than the general population. There can be significant differences in the prevalence, treatment and outcomes of chronic pain conditions across education levels, socioeconomic status, ethnicities and cultures (Blyth et al., 2001; Morales & Yong, 2021) Future research should replicate our work to determine if our findings are generalizable across more diverse samples. Third, questions were only assessed once. Future research should include longitudinal assessment across pain treatment for a more rigorous examination of how frequency/intensity of disordered eating behaviors change alongside change in pain symptoms. Relatedly, best practices for scale evaluation include tests of reliability, validity, and dimensionality (Boateng et al., 2018). While we were able to establish internal reliability and validity, we did not utilize a longitudinal structure that would allow us to evaluate test–retest reliability or dimensionality. Future research should continue to establish the psychometric soundness of these measures through confirmatory factor analysis and longitudinal samples. Additionally, these brief eating disorder measures may not capture the scope of various eating problems experienced by patients with chronic pain; however, no broader scale to assess eating problems for patients with chronic health conditions exists. This may be meaningful work of future studies. However, this present study is an important first step in validating brief screening measures to inform research and clinical practice decisions for patients with chronic pain.

## Conclusion

This study highlights the critical intersection of chronic pain and eating problems, demonstrating the utility of the EAT-8 and EDE-Q8 as valid screening tools for identifying disordered eating behaviors in English-speaking adults with chronic pain. Our findings establish these brief measures as reliable and construct-valid tools that distinguish eating behaviors based on eating disorder history and BMI. Despite these promising results, the study also reveals potential gaps in capturing the full scope of eating problems unique to chronic pain populations, emphasizing the need for more nuanced and context-specific tools. The strong association between chronic pain and disordered eating behaviors, alongside shared risk factors and mechanisms like central sensitization, underscores the importance of early identification and intervention to improve patient outcomes. Future research should expand on these findings by exploring qualitative methods, longitudinal analyses, and diverse populations to refine screening and intervention strategies. By advancing assessment methods, this work lays the foundation for integrating psychological and physical health approaches in chronic pain treatment, addressing the complex interplay of pain and disordered eating behaviors.

## Supplementary Information

Below is the link to the electronic supplementary material.

Supplementary file1 (DOCX 31 KB)

## Data Availability

The data that support the findings of this study are available upon reasonable request from the corresponding author.
