# Supplementary material for: Preliminary Validation of Two Brief Screening Measures for Eating Disorders in Adults with Chronic Pain
Source: J Clin Psychol Med Settings. 2025 Dec 28;33(1):109–18. doi: 10.1007/s10880-025-10111-2 (PMC13035618; doi:10.1007/s10880-025-10111-2)
Supplement: Supplementary file 1 — Supplementary file1 (DOCX 31 KB) [file 10880_2025_10111_MOESM1_ESM.docx]

**Supplementary Tables**

| **Table S1. Demographic Variables by Self-Reported Eating Disorder History** | | | | | |
| --- | --- | --- | --- | --- | --- |
|  |  | **No** | **Yes** | **Not Sure** | **Total** |
|  | | N (%) | N (%) | N (%) | N (%) |
| Age, mean (SD) | | 46.4 (14.2) | 39.1 (11.9) | 45.1 (14.8) |  |
| BMI, mean (SD) | | 31.6 (9.6) | 31.0 (7.0) | 34.7 (11.3) |  |
| Payer | |  |  |  |  |
|  | Commercial | 68 (51.9%) | 6 (37.5%) | 6 (46.2%) | 80 (50%) |
|  | Medicaid | 30 (22.9%) | 4 (25%) | 5 (38.5%) | 39 (24.4%) |
|  | Medicare | 30 (22.9%) | 6 (37.5%) | 2 (15.4%) | 38 (23.8%) |
|  | Other | 3 (2.3%) | 0 (0%) | 0 (0%) | 3 (1.9%) |
| Race | |  |  |  |  |
|  | White | 81 (85.3%) | 11 (91.7%) | 8 (100%) | 100 (87%) |
|  | Other | 14 (14.7%) | 1 (8.3%) | 0 (0%) | 15 (13%) |
| Gender | |  |  |  |  |
|  | Female | 73 (76%) | 12 (100%) | 7 (87.5%) | 92 (79.3%) |
|  | Other | 23 (24%) | 0 (0%) | 1 (12.5%) | 24 (20.7%) |
| Sex | |  |  |  |  |
|  | Female | 74 (78.7%) | 11 (100%) | 7 (87.5%) | 92 (81.4%) |
|  | Other | 20 (21.3%) | 0 (0%) | 1 (12.5%) | 21 (18.6%) |
| Sexual Preference | |  |  |  |  |
|  | Bisexual | 8 (8.5%) | 2 (16.7%) | 3 (37.5%) | 13 (11.4%) |
|  | Straight | 75 (79.8%) | 8 (66.7%) | 3 (37.5%) | 86 (75.4%) |
|  | Other | 11 (11.7%) | 2 (16.7%) | 2 (25%) | 15 (13.2%) |
| Education | |  |  |  |  |
|  | High School or less | 12 (12.5%) | 4 (33.3%) | 2 (25%) | 18 (15.5%) |
|  | Some College | 28 (29.2%) | 2 (16.7%) | 3 (37.5%) | 33 (28.4%) |
|  | Associate degree | 14 (14.6%) | 1 (8.3%) | 0 (0%) | 15 (12.9%) |
|  | Bachelor’s degree | 21 (21.9%) | 3 (25%) | 2 (25%) | 26 (22.4%) |
|  | Graduate degree | 21 (21.9%) | 2 (16.7%) | 1 (12.5%) | 24 (20.7%) |
| Marital Status | |  |  |  |  |
|  | Single | 27 (28.7%) | 7 (58.3%) | 2 (28.6%) | 36 (31.9%) |
|  | Married | 51 (54.3%) | 1 (8.3%) | 3 (42.9%) | 55 (48.7%) |
|  | Previously Married | 16 (17%) | 4 (33.3%) | 2 (28.6%) | 22 (19.5%) |
| Employment | |  |  |  |  |
|  | Full time | 27 (28.4%) | 2 (16.7%) | 2 (25%) | 31 (27%) |
|  | Part time | 12 (12.6%) | 1 (8.3%) | 1 (12.5%) | 14 (12.2%) |
|  | Retired | 12 (12.6%) | 0 (0%) | 1 (12.5%) | 13 (11.3%) |
|  | Homemaker | 4 (4.2%) | 0 (0%) | 2 (25%) | 6 (5.2%) |
|  | Student | 8 (8.4%) | 1 (8.3%) | 0 (0%) | 9 (7.8%) |
|  | STD or LTD | 6 (6.3%) | 0 (0%) | 0 (0%) | 6 (5.2%) |
|  | SSDI | 21 (22.1%) | 5 (41.7%) | 0 (0%) | 26 (22.6%) |
|  | Unemployed | 5 (5.3%) | 3 (25%) | 2 (25%) | 10 (8.7%) |
| Work - Pay | |  |  |  |  |
|  | 1 to 10 hours | 10 (10.5%) | 3 (25%) | 1 (12.5%) | 14 (12.2%) |
|  | 11 to 20 hours | 3 (3.2%) | 0 (0%) | 0 (0%) | 3 (2.6%) |
|  | 21 to 40 hours | 13 (13.7%) | 2 (16.7%) | 1 (12.5%) | 16 (13.9%) |
|  | > 40 hours | 21 (22.1%) | 1 (8.3%) | 2 (25%) | 24 (20.9%) |
|  | None | 48 (50.5%) | 6 (50%) | 4 (50%) | 58 (50.4%) |
| Work - Home | |  |  |  |  |
|  | 1 to 10 hours | 60 (63.8%) | 7 (58.3%) | 3 (37.5%) | 70 (61.4%) |
|  | 11 to 20 hours | 17 (18.1%) | 2 (16.7%) | 2 (25%) | 21 (18.4%) |
|  | 21 to 40 hours | 7 (7.4%) | 2 (16.7%) | 2 (25%) | 11 (9.6%) |
|  | > 40 hours | 5 (5.3%) | 1 (8.3%) | 1 (12.5%) | 7 (6.1%) |
|  | None | 5 (5.3%) | 0 (0%) | 0 (0%) | 5 (4.4%) |
| Work - School | |  |  |  |  |
|  | 1 to 10 hours | 14 (14.7%) | 1 (8.3%) | 0 (0%) | 15 (13%) |
|  | 11 to 20 hours | 4 (4.2%) | 0 (0%) | 0 (0%) | 4 (3.5%) |
|  | 21 to 40 hours | 4 (4.2%) | 0 (0%) | 0 (0%) | 4 (3.5%) |
|  | > 40 hours | 1 (1.1%) | 1 (8.3%) | 0 (0%) | 2 (1.7%) |
|  | None | 72 (75.8%) | 10 (83.3%) | 8 (100%) | 90 (78.3%) |
| Work - Volunteering | |  |  |  |  |
|  | 1 to 10 hours | 23 (24.2%) | 6 (50%) | 3 (37.5%) | 32 (27.8%) |
|  | None | 72 (75.8%) | 6 (50%) | 5 (62.5%) | 83 (72.2%) |
| Income | |  |  |  |  |
|  | Comfortable | 42 (43.8%) | 0 (0%) | 2 (25%) | 44 (37.9%) |
|  | Just enough | 29 (30.2%) | 4 (33.3%) | 3 (37.5%) | 36 (31%) |
|  | NOT enough | 25 (26%) | 8 (66.7%) | 3 (37.5%) | 36 (31%) |
| Pain Duration | |  |  |  |  |
|  | < 1 years | 3 (3.2%) | 1 (9.1%) | 0 (0%) | 4 (3.5%) |
|  | 1 to 3 years | 9 (9.5%) | 3 (27.3%) | 0 (0%) | 12 (10.5%) |
|  | 3 to 5 years | 12 (12.6%) | 1 (9.1%) | 1 (12.5%) | 14 (12.3%) |
|  | 5 to 10 years | 24 (25.3%) | 0 (0%) | 3 (37.5%) | 27 (23.7%) |
|  | > 10 years | 47 (49.5%) | 6 (54.5%) | 4 (50%) | 57 (50%) |

| **Table S4. Demographic Variables by BMI Category** | | | | | | |
| --- | --- | --- | --- | --- | --- | --- |
|  |  | **Underweight** | **Healthy** | **Overweight** | **Obese** | **Total** |
|  | | N (%) | N (%) | N (%) | N (%) | N (%) |
| Age, mean (SD) | | 37.7 (14.8) | 44.7 (17.4) | 45.4 (16) | 46.9 (11.1) |  |
| Payer | |  |  |  |  |  |
|  | Commercial | 3 (50%) | 19 (51.4%) | 20 (54.1%) | 39 (48.1%) | 81 (50.3%) |
|  | Medicaid | 2 (33.3%) | 9 (24.3%) | 7 (18.9%) | 20 (24.7%) | 38 (23.6%) |
|  | Medicare | 1 (16.7%) | 7 (18.9%) | 10 (27%) | 21 (25.9%) | 39 (24.2%) |
|  | Other | 0 (0%) | 2 (5.4%) | 0 (0%) | 1 (1.2%) | 3 (1.9%) |
| Race | |  |  |  |  |  |
|  | White | 3 (100%) | 24 (85.7%) | 22 (91.7%) | 52 (86.7%) | 101 (87.8%) |
|  | Other | 0 (0%) | 4 (14.3%) | 2 (8.3%) | 8 (13.3%) | 14 (12.2%) |
| Gender | |  |  |  |  |  |
|  | Female | 3 (100%) | 22 (78.6%) | 18 (72%) | 49 (81.7%) | 92 (79.3%) |
|  | Other | 0 (0%) | 6 (21.4%) | 7 (28%) | 11 (18.3%) | 24 (20.7%) |
| Sex | |  |  |  |  |  |
|  | Female | 2 (100%) | 21 (77.8%) | 18 (75%) | 51 (85%) | 92 (81.4%) |
|  | Other | 0 (0%) | 6 (22.2%) | 6 (25%) | 9 (15%) | 21 (18.6%) |
| Sexual Preference | |  |  |  |  |  |
|  | Bisexual | 0 (0%) | 2 (7.4%) | 2 (8%) | 9 (15.3%) | 13 (11.4%) |
|  | Straight | 2 (66.7%) | 20 (74.1%) | 18 (72%) | 46 (78%) | 86 (75.4%) |
|  | Other | 1 (33.3%) | 5 (18.5%) | 5 (20%) | 4 (6.8%) | 15 (13.2%) |
| Education | |  |  |  |  |  |
|  | High School or less | 0 (0%) | 4 (14.3%) | 5 (20%) | 9 (15%) | 18 (15.5%) |
|  | Some College | 0 (0%) | 6 (21.4%) | 3 (12%) | 23 (38.3%) | 32 (27.6%) |
|  | Associate degree | 1 (33.3%) | 2 (7.1%) | 2 (8%) | 10 (16.7%) | 15 (12.9%) |
|  | Bachelor degree | 0 (0%) | 9 (32.1%) | 8 (32%) | 9 (15%) | 26 (22.4%) |
|  | Graduate degree | 2 (66.7%) | 7 (25%) | 7 (28%) | 9 (15%) | 25 (21.6%) |
| Marital Status | |  |  |  |  |  |
|  | Single | 2 (66.7%) | 11 (39.3%) | 13 (54.2%) | 28 (48.3%) | 54 (47.8%) |
|  | Married | 0 (0%) | 11 (39.3%) | 9 (37.5%) | 17 (29.3%) | 37 (32.7%) |
|  | Previously Married | 1 (33.3%) | 6 (21.4%) | 2 (8.3%) | 13 (22.4%) | 22 (19.5%) |
| Employment | |  |  |  |  |  |
|  | Full time | 2 (66.7%) | 9 (32.1%) | 8 (32%) | 12 (20.3%) | 31 (27%) |
|  | Part time | 0 (0%) | 2 (7.1%) | 5 (20%) | 7 (11.9%) | 14 (12.2%) |
|  | Retired | 0 (0%) | 3 (10.7%) | 4 (16%) | 6 (10.2%) | 13 (11.3%) |
|  | Homemaker | 1 (33.3%) | 0 (0%) | 0 (0%) | 5 (8.5%) | 6 (5.2%) |
|  | Student | 0 (0%) | 4 (14.3%) | 3 (12%) | 2 (3.4%) | 9 (7.8%) |
|  | STD or LTD | 0 (0%) | 2 (7.1%) | 0 (0%) | 4 (6.8%) | 6 (5.2%) |
|  | SSDI | 0 (0%) | 6 (21.4%) | 3 (12%) | 17 (28.8%) | 26 (22.6%) |
|  | Unemployed | 0 (0%) | 2 (7.1%) | 2 (8%) | 6 (10.2%) | 10 (8.7%) |
| Work - Pay | |  |  |  |  |  |
|  | 1 to 10 hours | 0 (0%) | 3 (10.7%) | 6 (24%) | 5 (8.5%) | 14 (12.2%) |
|  | 11 to 20 hours | 0 (0%) | 1 (3.6%) | 2 (8%) | 0 (0%) | 3 (2.6%) |
|  | 21 to 40 hours | 0 (0%) | 5 (17.9%) | 2 (8%) | 9 (15.3%) | 16 (13.9%) |
|  | > 40 hours | 2 (66.7%) | 5 (17.9%) | 7 (28%) | 10 (16.9%) | 24 (20.9%) |
|  | None | 1 (33.3%) | 14 (50%) | 8 (32%) | 35 (59.3%) | 58 (50.4%) |
| Work - Home | |  |  |  |  |  |
|  | 1 to 10 hours | 3 (100%) | 18 (66.7%) | 15 (60%) | 34 (57.6%) | 70 (61.4%) |
|  | 11 to 20 hours | 0 (0%) | 4 (14.8%) | 6 (24%) | 10 (16.9%) | 20 (17.5%) |
|  | 21 to 40 hours | 0 (0%) | 0 (0%) | 2 (8%) | 10 (16.9%) | 12 (10.5%) |
|  | > 40 hours | 0 (0%) | 2 (7.4%) | 2 (8%) | 3 (5.1%) | 7 (6.1%) |
|  | None | 0 (0%) | 3 (11.1%) | 0 (0%) | 2 (3.4%) | 5 (4.4%) |
| Work - School | |  |  |  |  |  |
|  | 1 to 10 hours | 0 (0%) | 3 (10.7%) | 6 (25%) | 6 (10%) | 15 (13%) |
|  | 11 to 20 hours | 0 (0%) | 2 (7.1%) | 1 (4.2%) | 1 (1.7%) | 4 (3.5%) |
|  | 21 to 40 hours | 0 (0%) | 1 (3.6%) | 1 (4.2%) | 2 (3.3%) | 4 (3.5%) |
|  | > 40 hours | 0 (0%) | 1 (3.6%) | 1 (4.2%) | 0 (0%) | 2 (1.7%) |
|  | None | 3 (100%) | 21 (75%) | 15 (62.5%) | 51 (85%) | 90 (78.3%) |
| Work - Volunteering | |  |  |  |  |  |
|  | 1 to 10 hours | 1 (33.3%) | 11 (39.3%) | 5 (20.8%) | 15 (25%) | 32 (27.8%) |
|  | None | 2 (66.7%) | 17 (60.7%) | 19 (79.2%) | 45 (75%) | 83 (72.2%) |
| Income | |  |  |  |  |  |
|  | Comfortable | 2 (66.7%) | 13 (46.4%) | 13 (52%) | 16 (26.7%) | 44 (37.9%) |
|  | Just enough | 1 (33.3%) | 7 (25%) | 8 (32%) | 19 (31.7%) | 35 (30.2%) |
|  | NOT enough | 0 (0%) | 8 (28.6%) | 4 (16%) | 25 (41.7%) | 37 (31.9%) |
| Pain Duration | |  |  |  |  |  |
|  | < 1 years | 0 (0%) | 0 (0%) | 2 (8.3%) | 2 (3.3%) | 4 (3.5%) |
|  | 1 to 3 years | 0 (0%) | 4 (14.8%) | 0 (0%) | 8 (13.3%) | 12 (10.5%) |
|  | 3 to 5 years | 2 (66.7%) | 5 (18.5%) | 4 (16.7%) | 3 (5%) | 14 (12.3%) |
|  | 5 to 10 years | 0 (0%) | 7 (25.9%) | 6 (25%) | 14 (23.3%) | 27 (23.7%) |
|  | > 10 years | 1 (33.3%) | 11 (40.7%) | 12 (50%) | 33 (55%) | 57 (50%) |
